# Respiratory Function and Oxidative Stress in Smoking Zinc Smelter Workers Exposed to Lead

**DOI:** 10.3390/jcm14228198

**Published:** 2025-11-19

**Authors:** Tomasz Chwalba, Marta Wąsik, Michał Dobrakowski, Artur Chwalba, Malgorzata Jekielek, Aleksandra Kasperczyk, Jolanta Zalejska-Fiolka, Francesco Bellanti, Rafał J. Bułdak, Beata Maksym, Sławomir Kasperczyk

**Affiliations:** 1 Department of Anathomy, Faculty of Medical Sciences in Zabrze, Medical University of Silesia in Katowice, Jordana 19, 41-808 Zabrze, Poland; 2 Department of Clinical Biochemistry and Laboratory Diagnostics, Institute of Medicine, Opole University, Oleska 48, 45-052 Opole, Poland; 3 Department of Radiology and Radiodiagnostics, Faculty of Medical Sciences in Zabrze, Medical University of Silesia in Katowice, 3-go Maja 13-15, 41-800 Zabrze, Poland; 4 Department of Pharmacology, Faculty of Medical Sciences in Zabrze, Medical University of Silesia in Katowice, Jordana 19, 41-808 Zabrze, Poland; 5 Department of Physiotherapy, Faculty of Health Sciences, Jagiellonian University Collegium Medicum, 31-008 Cracow, Poland; 6 Department of Biochemistry, Faculty of Medical Sciences in Zabrze, Medical University of Silesia in Katowice, Jordana 19, 41-808 Zabrze, Poland; 7 Department of Medical and Surgical Sciences, University of Foggia, Viale Pinto 1, 71122 Foggia, Italy

**Keywords:** occupational exposure, toxic metals, occupational disease

## Abstract

**Background/Objectives:** Lead, a toxic heavy metal, is widely recognized as a hazardous environmental contaminant capable of disrupting physiological homeostasis by altering stress response mechanisms and impairing pulmonary function. A comparable detrimental factor is tobacco smoking, which represents one of the most prevalent addictions worldwide. The presented study aimed to evaluate the combined impact of cigarette smoking and occupational lead exposure on selected oxidative stress biomarkers and pulmonary function parameters. **Methods:** 453 male employees working in a zinc smelter were recruited for participation in the study. Participants were subsequently divided into two groups: current smokers (*n* = 209) and former smokers (*n* = 244). Each group was then further subdivided according to blood lead concentration into subgroups with high (>35 μg/dL) and low (<35 μg/dL) lead levels. Venous blood samples were collected for biochemical analysis of oxidative stress parameters, including total oxidant status (TOS), malondialdehyde (MDA), protein thiol content (PSH), total antioxidant capacity (TAC), and the oxidative stress index (OSI). In addition, spirometric evaluation was conducted. **Results:** Former smokers demonstrated significantly more favorable oxidative stress profiles and superior spirometric outcomes compared with current smokers. No statistically significant associations were observed between lead exposure levels and either biochemical or spirometric parameters. **Conclusions:** Cigarette smoking appears to exert a stronger adverse influence on oxidative balance and pulmonary function than occupational lead exposure.

## 1. Introduction

Lead, as a heavy metal, is classified as a toxic element with substantial adverse effects on human health. There are multiple routes of exposure, including ingestion of contaminated food or water, inhalation of polluted air, or exposure to industrial dust in areas with intensive metallurgical activity [1,2]. Airborne lead particles are particularly hazardous to the respiratory tract, as they may accumulate in lung tissue, causing structural damage and impairing respiratory efficiency [3,4]. Beyond its direct toxic effects on the respiratory system, lead exposure disturbs systemic homeostasis. One of the key mechanisms underlying this disturbance is oxidative stress, defined as an imbalance between oxidant production and antioxidant defense, resulting in excessive generation of reactive oxygen species (ROS) and insufficient neutralization of these radicals. Chronic oxidative stress contributes to impaired tissue regeneration and accelerated cellular aging [5,6,7,8]. Biochemical assays enable the detection of molecular markers of oxidative stress, whose elevated concentrations indicate redox imbalance within the organism. Besides lead exposure, cigarette smoking constitutes another major factor contributing to oxidative stress and respiratory dysfunction. Tobacco smoke contains numerous toxic compounds, many of which are reactive oxygen and nitrogen species (ROS and RNS), which can damage critical cellular components, including proteins, lipids, and nucleic acids. Sustained exposure to these reactive species promotes oxidative stress and chronic inflammation, leading to conditions such as asthma, chronic obstructive pulmonary disease (COPD), and lung cancer [9,10]. The aim of the presented study was to assess the effects of occupational lead exposure on respiratory function and oxidative stress markers among zinc smelter workers who smoke or have smoked in the past.

## 2. Materials and Methods

The study protocol was reviewed and approved by the Bioethics Committee of the Medical University of Silesia in Katowice (No. KNW/022/KB1/108/14). All procedures were conducted in accordance with the ethical principles of the Declaration of Helsinki.

### 2.1. Participants

In total, 453 male employees of the zinc smelter in Miasteczko Śląskie, Poland, were enrolled in the study. The inclusion criteria comprised current or former cigarette smoking—all participants reported the use of conventional tobacco products. Eligible participants were required to have been employed at the smelter for at least one year, and to have started their employment there within the last five years. All participants were free of comorbidities and did not report any acute or chronic illnesses during the study period. Based on the applicable labor law, the permissible concentration of lead in biological material is 50 µg Pb/100 mL of blood. The patients included in the study did not exceed the legally required blood lead concentration.

At baseline, participants were divided into two groups: current smokers and former smokers. Each of these groups was subsequently subdivided according to blood lead concentration into low (<35 μg/dL) and high (>35 μg/dL) exposure subgroups. Demographic and occupational data, including age, body mass index (BMI), years of employment, smoking duration, and the number of cigarettes smoked per day (current and past), were collected. The pack-year index was calculated as the number of cigarette packs smoked per day multiplied by the total years of smoking. Baseline characteristics did not differ significantly between study groups, and detailed data are presented in Table 1.

Prior to participation, all patients provided written informed consent after receiving a full explanation of the study objectives and procedures. Participants were informed that they could withdraw from the study at any stage without providing a reason. To ensure confidentiality, each participant was assigned a unique identification code for anonymized data analysis.

### 2.2. Data Collection and Research Procedure

The study population consisted of male employees occupationally exposed to heavy metals in various departments of the zinc and lead smelter located in Miasteczko Śląskie, Poland. Inclusion criteria required a minimum of one year of continuous employment. All participants were free of comorbidities and did not report any acute or chronic illnesses during the study period.

Venous blood samples (20 mL) were collected under standardized conditions by trained medical personnel. 10 mL were drawn into ethylenediaminetetraacetic acid (EDTA)-containing tubes for hematological and biochemical analyses, while the remaining 10 mL were collected into plain tubes for serum separation. Samples were properly labeled, stored under controlled conditions, and processed promptly to maintain biological integrity.

Oxidative stress parameters determined in serum included total oxidant status (TOS), malondialdehyde (MDA), protein thiol content (PSH), total antioxidant capacity (TAC), and the oxidative stress index (OSI). These biomarkers are increasingly utilized to assess in vivo redox homeostasis; however, their interpretation is influenced by biological variability and methodological differences. Despite these limitations, they provide valuable insights into oxidative processes in occupational exposure studies [11,12].

All laboratory analyses were performed at the Department of Biochemistry, Silesian Medical University, Zabrze, Poland. Spirometric examinations were conducted using a Spirolab New Spirometer with an integrated pulse oximeter (MIR, Rome, Italy), following the American Thoracic Society (ATS) recommendations. Parameters evaluated included forced vital capacity (FVC), forced expiratory volume in one second (FEV_1_), FEV_1_/FVC ratio, and peak expiratory flow (PEF).

#### 2.2.1. Measurement of Blood Lead Concentration

Blood lead (PbB) and zinc protoporphyrin (ZPP) levels were used as biomarkers of occupational lead exposure, determined according to standardized analytical procedures [13]. PbB concentration was quantified using graphite furnace atomic absorption spectrometry (GFAAS) on an ICE 3400 spectrometer (Thermo Fisher Scientific, Waltham, MA, USA) at a wavelength of 283.3 nm, with results expressed in micrograms per deciliter (μg/dL).

ZPP concentrations were measured fluorometrically using the Aviv HF 206 hematofluorometer (Aviv Biomedical, Lakewood, NJ, USA). The fluorescence intensity (excitation at 415 nm, emission at 596 nm) was normalized to hemoglobin absorbance, and results were expressed as micrograms of ZPP per gram of hemoglobin (μg/g Hb).

#### 2.2.2. Measurement of Total Oxidant Status (TOS)

Serum TOS was determined according to the Erel method [14], which measures the oxidative potential of serum components. In this assay, oxidants convert ferrous ions (Fe^2+^) to ferric ions (Fe^3+^) under acidic conditions; ferric ions form a colored complex with xylenol orange, the absorbance of which (560 nm) is directly proportional to total oxidant levels. Hydrogen peroxide served as the calibration standard, and results were expressed as micromoles per liter (μmol/L).

#### 2.2.3. Measurement of Total Antioxidant Capacity (TAC)

Serum TAC was quantified using Erel’s colorimetric method [15], which evaluates the ability of antioxidants in serum to neutralize free radicals. The method used the ABTS radical cation (2,2′-azinobis-(3-ethylbenzothiazoline-6-sulfonic acid)), and the decrease in its blue-green coloration (660 nm) is measured spectrophotometrically. Trolox, a water-soluble vitamin E analogue, was used as the standard, and TAC values were expressed in millimoles per liter (mmol/L).

#### 2.2.4. Measurement of Malondialdehyde (MDA)

MDA levels in serum and erythrocytes were determined using a modified thiobarbituric acid (TBA) assay based on the method of Ohkawa et al. [16]. The technique measures fluorescent MDA–TBA adducts, formed as products of lipid peroxidation. To enhance assay specificity, sodium sulfate and butylated hydroxytoluene (BHT) were added to prevent artificial oxidation during sample handling. Measurements were performed spectrofluorometrically (excitation 515 nm, emission 522 nm) and compared to a standard curve generated using 1,1,3,3-tetraethoxypropane. Results were expressed as μmol/L in serum and nmol/g Hb in erythrocytes.

#### 2.2.5. Measurement of Oxidative Stress Index (OSI)

The oxidative stress index (OSI) was calculated as the ratio of TOS to TAC, expressed as a percentage, following appropriate unit normalization. This composite indicator provides a comprehensive assessment of the redox balance, where higher OSI values indicate greater oxidative burden and antioxidant depletion.

#### 2.2.6. Measurement of Protein Thiol Content (PSH)

Serum protein thiol content was measured using the Koster et al. method [17], based on the reaction of the sulfhydryl groups with 5,5′-dithiobis-(2-nitrobenzoic acid) (DTNB). The reduction in DTNB produces the yellow 5-thio-2-nitrobenzoate anion (TNB), measured at 412 nm using an automated biochemical analyzer (PerkinElmer, Waltham, MA, USA). Values were normalized to total protein concentration and expressed as μmol/g protein.

### 2.3. Statistical Analysis

Statistical analyses were conducted using PS IMAGO PRO 10 (Predictive Solutions, Krakow, Poland) and STATISTICA version 13.3 software (TIBCO Software Inc., Palo Alto, CA, USA) on a Windows 10 (64-bit) operating system. Data normality was assessed using the Shapiro–Wilk test. For normally distributed variables, results were expressed as means ± standard deviations (SD), and independent-samples Student’s *t*-tests were applied to assess between-group differences. The level of statistical significance was set at *p* < 0.05.

## 3. Results

### 3.1. Oxidative Stress Parameters

A detailed summary of the obtained biochemical results is presented in Table 2.

#### 3.1.1. Comparison Between Former and Current Smokers

When comparing former smokers and current smokers, three parameters demonstrated statistically significant differences. The mean concentration of protein thiols (PSH) was higher among former smokers (314.40 μmol/L, SD = 44.03) compared with current smokers (300.70 μmol/L, SD = 43.94). Similarly, total antioxidant capacity (TAC) was significantly greater in former smokers (1.16 mmol/L, SD = 0.13) than in current smokers (1.12 mmol/L, SD = 0.11). The oxidative stress index (OSI) also differed significantly, with higher values observed among current smokers (1.08%, SD = 0.87) compared with former smokers (1.00%, SD = 0.94). No statistically significant differences were noted in total oxidant status (TOS) or malondialdehyde (MDA) concentrations between the two groups.

#### 3.1.2. Comparison of Results Between Subgroups with Low and High Lead Levels

When comparing participants with low (<35 μg/dL) and high (>35 μg/dL) blood lead concentrations, a statistically significant difference in MDA concentration was found only among former smokers. In this subgroup, MDA levels were 2.94 μmol/L (SD = 2.02) in those with low lead levels and 3.32 μmol/L (SD = 1.39) in those with high lead levels. Although other oxidative stress parameters (TOS, TAC, OSI, PSH) did not differ significantly, a trend toward higher TOS, TAC, and OSI values and lower PSH levels was observed among individuals with elevated blood lead concentrations. Among current smokers, no statistically significant differences were found between the high and low lead exposure groups. Mean TOS, MDA, and OSI values were slightly higher in participants with low lead levels, whereas TAC and PSH values tended to be higher in those with higher lead concentrations.

### 3.2. Spirometry Results

Detailed data are provided in Table 3.

#### 3.2.1. Comparison of Past and Current Smokers’ Results

When analyzing spirometric parameters according to smoking status, statistically significant differences were observed in five indices. Current smokers exhibited a significantly lower mean forced vital capacity (FVC) than former smokers (4.27 L, SD = 0.99 vs. 4.43 L, SD = 0.91, respectively). Likewise, former smokers achieved higher mean and recent values of forced expiratory volume in one second (FEV_1_): 3.97 L (SD = 0.79) and 4.22 L (SD = 2.14) compared with 3.83 L (SD = 0.95) and 3.91 L (SD = 0.87) in current smokers. Peak expiratory flow (PEF) results also differed significantly: former smokers demonstrated superior mean and recent PEF values (9.24 L/s, SD = 2.20; 9.54 L/s, SD = 1.92) compared with current smokers, whose mean PEF values did not exceed 9 L/s in either assessment.

#### 3.2.2. Comparison of Results Between Subgroups with Low and High Lead Levels

The analysis also examined whether there were differences in spirometry results comparing low and high lead levels between smokers and former smokers. After examining the results, no statistically significant differences were found between former smokers divided into low and high lead levels, and between current smokers divided by lead level.

## 4. Discussion

The presented study was undertaken as a continuation of the authors’ long-standing research interests in biochemistry, internal medicine, and laboratory diagnostics. A particularly important aspect of integrating these three scientific disciplines is the investigation of exposure to potentially toxic environmental and occupational factors. The Silesian region of Poland, from which the authors originate, represents one of the most industrialized areas in the country, characterized by extensive heavy-industry activity and environmental contamination. Among workers employed in metal processing facilities such as steel and zinc smelters, chronic exposure to lead remains a key determinant of occupational health outcomes. Previous research conducted by the authors demonstrated that lead exposure may impair respiratory function and increase systemic oxidative stress. In the presented study, oxidative stress parameters and spirometric indices were used as objective indicators of redox imbalance and pulmonary performance, respectively. The authors aimed to determine whether concurrent exposure to lead and cigarette smoking would result in an additive or synergistic deterioration of these parameters, or whether lead toxicity alone would predominate. The results demonstrated that cigarette smoking exerted a more deleterious effect on the human body than occupational lead exposure, as evidenced by elevated oxidative stress levels and reduced lung function. The detrimental influence of cigarette smoking on respiratory performance has been comprehensively documented in the literature, and numerous investigators have also sought to evaluate the combined or interactive effects of tobacco use and heavy-metal exposure, both in occupational settings and environmentally exposed populations. In a study by Mistry et al., 105 male participants were examined, of whom 51 were active smokers and the remainder reported never smoking. The mean age of the study population exceeded 41 years. Spirometric analysis included forced vital capacity (FVC), peak expiratory flow rate (PEFR), forced expiratory flow (FEF), forced expiratory volume in one second (FEV_1_), and the FEV_1_/FVC ratio. Among smokers, all measured indices were significantly reduced compared with non-smokers. Moreover, a clear dose–response relationship was identified, showing that the higher the daily cigarette consumption and the longer the smoking duration, the greater the impairment in spirometric values [18]. Similarly, Isah et al. investigated 200 individuals (150 smokers and 50 never-smokers) with a mean age of 34.27 ± 8.91 years among smokers and 35.08 ± 10.35 years among non-smokers. Data collection involved questionnaire assessment and spirometry performed with a Clement Clarke spirometer. In participants exhibiting respiratory abnormalities, spirometry was repeated following bronchodilator administration. Consistent with the findings of the present investigation, both FEV_1_ and FEV_1_/FVC ratios were significantly lower in smokers compared with never-smokers. Participants in the authors’ study were approximately ten years older on average, which may have contributed to the cumulative adverse effects of lead exposure and smoking. Nonetheless, the general pattern of spirometric decline remained consistent across studies [19]. Kumar et al. also examined restricted airflow and smoking-related alterations in pulmonary function. Their cohort comprised 300 individuals aged 25–60 years, divided equally into smokers and non-smokers. The mean age of smokers was 49.98 ± 11.43 years, whereas that of non-smokers was 47.25 ± 12.82 years. Smoking intensity was classified into three categories according to a modified smoking index: low (≤100 cigarettes), moderate (≤300 cigarettes), and heavy (>300 cigarettes). This index, a variation of the traditional pack-year measure, accounts for the total number of cigarettes rather than the number of packs consumed. In all spirometric variables—FVC, FEV_1_, PEFR, and FEF 25–75%—smokers exhibited significantly lower mean values than non-smokers. Although the study population was slightly older than in the current work, the observed downward trend in pulmonary efficiency among smokers remained comparable, reinforcing the strong negative impact of tobacco use on respiratory function [20]. The relationship between lead exposure and respiratory impairment has also been intensively explored in recent literature. A particularly noteworthy contribution was presented by Wei Wei et al., who evaluated the combined effects of lead exposure and genetic variability on oxidative stress and lung function. Their four-year longitudinal study included 1243 coking-plant workers with documented exposure to lead and cadmium. Lead concentrations were determined in urine samples, and the analysis encompassed 2664 single-nucleotide polymorphisms (SNPs) across 345 genes potentially linked to oxidative stress. The authors reported that higher urinary lead concentrations were associated with a significant decline in FEV_1_. Moreover, the NQO1 rs2917670 polymorphism appeared to modulate the effect of lead exposure on FEV_1_ changes. Another important finding indicated that high lead exposure correlated with increased expression of the oxidative-stress-related polymorphism 8-iso-PGF_2_α. Such molecular-level investigations underscore the importance of integrating genetic and environmental data to elucidate individual susceptibility to oxidative injury. The findings by Wei Wei et al. open new perspectives in occupational medicine, toxicogenomics, and personalized risk assessment [21]. In a separate study, Kuo-Jung Ho and colleagues evaluated oxidative stress markers such as oxidized low-density lipoprotein (OxLDL) and thiobarbituric acid-reactive substances (TBARS) in workers exposed to lead. Both cigarette smoking and lead exposure were considered potential contributors to systemic oxidative stress. The researchers noted that there are few studies examining the combined effects of smoking, lead exposure, and oxidative disorders. Their cohort included 267 employees (86 smokers and 181 non-smokers), and results demonstrated that smokers exhibited significantly higher mean blood-lead concentrations (22.11 ± 15.19 µg/dL) than non-smokers (7.97 ± 10.42 µg/dL). Additionally, oxidative stress biomarkers were markedly elevated among smokers: TBARS levels were 2.29 ± 0.56 nmol/L versus 1.95 ± 0.35 nmol/L, and OxLDL concentrations were 59.86 ± 14.57 mg/dL versus 53.76 ± 11.25 mg/dL in non-smokers. Both differences reached statistical significance [22]. The authors highlighted the critical importance of smoking cessation in occupational environments with ongoing lead exposure, consistent with the presented study’s conclusion that tobacco smoking constitutes a more potent determinant of oxidative and pulmonary dysfunction than lead exposure alone.

Despite its strengths, the current study is subject to several limitations. In future research, it would be advisable to include additional occupational variables, such as daily and weekly working hours, shift schedules, and irregular work patterns, all of which may contribute to increased oxidative burden. Another potentially valuable direction would involve distinguishing participants according to the type of cigarettes used—traditional tobacco products versus electronic cigarettes—given the distinct chemical profiles and toxicological impacts of these products. Furthermore, limitations associated with the selected oxidative-stress biomarkers (TAC, TOS, MDA, PSH) should be acknowledged. Although these indices provide meaningful insights into systemic redox balance, they are influenced by numerous biological and methodological factors. For instance, total antioxidant capacity (TAC) and total oxidant status (TOS) represent global measures that may vary depending on metabolic state, nutritional status, or analytical methodology. Moreover, the absence of universally standardized reference ranges and assay protocols complicates inter-study comparisons [12,23,24]. Consequently, some degree of interpretative uncertainty must be accepted when evaluating these data. Nonetheless, the study underscores the necessity of continued development in the field of oxidative-stress biomarker research and its integration into molecular toxicology and occupational health sciences.

## 5. Conclusions

Patients who were current smokers exhibited significantly higher levels of oxidative stress, as assessed by PSH and TAC measurements. Blood lead concentrations did not produce statistically significant differences in oxidative stress markers. These findings suggest that cigarette smoking may represent a stronger contributor to oxidative stress than lead exposure.

In spirometric assessment, current smokers demonstrated lower FVC, FEV_1_, and PEF values compared with individuals who had discontinued smoking, irrespective of blood lead levels. This indicates that smoking is a more potent factor impairing pulmonary function.

## 6. Clinical Implications

The differences observed between study participants who smoked and those who did not indicate that the use of tobacco products contributes to oxidative stress and impairments in lung function to a greater extent than occupational lead exposure. These findings underscore the need for preventive strategies aimed at encouraging individuals—particularly those employed in heavy industry—to stop smoking.

In the context of occupational health care, increased attention should be directed toward patients who smoke, as they are exposed to higher levels of oxidative stress and greater pulmonary injury compared with non-smokers. Routine preventive assessments may facilitate earlier detection of pathological changes and enable the initiation of less costly or shorter treatment modalities that do not require absence from work.

Future research should aim to achieve a more comprehensive understanding of the mechanisms underlying oxidative stress and pulmonary damage, taking into consideration factors such as genetic predispositions and chronic diseases within the studied population.

## Figures and Tables

**Table 1:** Characteristics of the study participants.

Parameter	Current Smokers *n* = 209					Former Smokers *n* = 244					Between Groups
	Low Blood Lead Concentration Pb < 35 μg/dL *n* = 106		High Blood Lead Concentration Pb > 35 μg/dL *n* = 103		Between Groups	Low Blood Lead Concentration Pb < 35 μg/dL *n* = 106		High Blood Lead Concentration Pb > 35 μg/dL *n* = 103		Between Groups	
Parameter	Mean	SD	Mean	SD	*p*	Mean	SD	Mean	SD	*p*	*p*
Age	42.60	10.01	44.53	9.10	0.15	43.40	10.00	44.14	8.60	0.55	0.81
BMI	27.20	4.20	26.60	4.00	0.31	28.20	3.60	28.50	4.10	0.54	0.00 *
Years of work	16.00	10.00	17.00	9.00	0.21	26.00	8.00	27.00	8.00	0.49	0.69
Number of cigarettes smoked currently	12.57	6.33	13.37	7.18	0.39	-	-	-	-	-	-
Number of cigarettes smoked before quitting	-	-	-	-	-	12.38	3.99	13.31	4.35	0.08	-
Number of years of smoking	17.35	8.96	20.05	8.71	0.03 *	12.50	7.86	12.97	7.60	0.63	0.00 *
Pack-year index	10.41	8.68	12.87	11.56	0.08	7.90	6.38	8.89	7.63	0.27	0.00 *
Blood lead concentration [μg/dL]	26.05	8.90	41.30	6.30	0.00 *	25.30	8.20	40.7	6.90	0.00 *	0.31

* statistically significant, SD—standard deviation.

**Table 2:** Oxidative Stress Parameters.

Parameter	Current Smokers *n* = 209							Former Smokers *n* = 244							*p*-Between Groups
	Total *n* = 209		Low Blood Lead Concentration Pb < 35 μg/dL *n* = 106		High Blood Lead Concentration Pb > 35 μg/dL *n* = 103		Between Groups	Total *n* = 244		Low Blood Lead Concentration Pb < 35 μg/dL *n* = 106		High Blood Lead Concentration Pb > 35 μg/dL *n* = 103		Between Groups	
Parameter	Mean	SD	Mean	SD	Mean	SD	*p*	Mean	SD	Mean	SD	Mean	SD	*p*	*p*
TOS [μmol/L]	11.01	4.25	11.26	4.57	10.73	3.86	0.43	10.91	5.79	10.79	6.29	11.08	5.13	0.73	0.86
MDA [μmol/L]	3.00	1.39	3.09	1.63	2.96	1.97	0.52	3.10	1.78	2.94	2.02	3.32	1.39	0.01 *	0.65
PSH [μmol/L]	300.70	43.94	299.1	44.21	302.40	43.82	0.63	314.40	44.03	315.30	46.26	313.30	41.27	0.76	0.00 *
TAC [mmol/L]	1.12	0.11	1.12	0.11	1.13	0.12	0.64	1.16	0.13	1.15	0.15	1.17	0.11	0.31	0.00 *
OSI [%]	1.08	0.87	1.11	0.93	1.04	0.80	0.59	1.01	0.94	1.04	1.18	0.97	0.49	0.31	0.01 *

* statistically significant, SD—standard deviation.

**Table 3:** Spirometry results.

Parameter	Current Smokers *n* = 209							Former Smokers *n* = 244							*p*-Between Groups
	Total *n* = 209		Low Blood Lead Concentration Pb < 35 μg/dL *n* = 106		High Blood Lead Concentration Pb > 35 μg/dL *n* = 103		Between Groups	Total *n* = 244		Low Blood Lead Concentration Pb < 35 μg/dL *n* = 106		High Blood Lead Concentration Pb > 35 μg/dL *n* = 103		Between Groups	
Parameter	Mean	SD	Mean	SD	Mean	SD	*p*	Mean	SD	Mean	SD	Mean	SD	*p*	*p*
FVC_Lr_ [L]	4.00	1.07	4.00	1.15	4.00	0.97	0.15	4.00	1.00	4.00	0.98	4.00	1.03	0.55	0.23
FVC_M_ [L]	4.27	0.99	4.35	1.07	4.18	0.89	0.22	4.43	0.91	4.45	0.87	4.40	0.95	0.63	0.02 *
FEV1_Lr_ [L]	3.83	0.95	3.92	0.99	3.75	0.91	0.21	3.97	0.79	4.02	0.79	3.92	0.79	0.32	0.04 *
FEV1_M_ [L]	3.91	0.87	3.99	0.90	3.83	0.82	0.17	4.22	2.14	4.12	0.70	4.32	2.99	0.47	0.00 *
FEV1/FVC_Lr_ [%]	92.50	6.90	92.4	6.50	92.7	7.30	0.82	100.50	78.40	101.20	81.3	99.6	75.50	0.87	0.15
FEV1/FVC_M_ [%]	92.40	6.39	92.62	6.36	92.20	6.44	0.65	98.27	59.99	96.96	40.91	99.00	75.37	0.67	0.16
PEF_Lr_ [L/s]	8.53	2.20	92.50	17.76	90.50	18.82	0.44	9.24	2.20	9.36	2.03	9.12	2.37	0.38	0.00 *
PEF_M_ [L/s]	8.96	2.71	8.57	2.02	8.48	2.39	0.76	9.54	1.92	9.55	1.68	9.53	2.16	0.05	0.01 *

* statistically significant, SD—standard deviation, Lr—last result, M—mean.

## Data Availability

The data collected from patients are stored anonymously in databases. Further inquiries can be directed to the corresponding author.
